# Current Trends in Cell-Free DNA Applications. Scoping Review of Clinical Trials

**DOI:** 10.3390/biology10090906

**Published:** 2021-09-13

**Authors:** Robert Stawski, Emilia Stec-Martyna, Adam Chmielecki, Dariusz Nowak, Ewelina Perdas

**Affiliations:** 1 Department of Clinical Physiology, Medical University of Lodz, 92-215 Lodz, Poland; dariusz.nowak@umed.lodz.pl; 2 Central Scientific Laboratory, Medical University of Lodz, 6/8 Mazowiecka St., 92-215 Lodz, Poland; emilia.stec-martyna@umed.lodz.pl; 3 Sport Centre of the Medical University of Lodz, Medical University of Lodz, 92-215 Lodz, Poland; adam.chmielecki@umed.lodz.pl; 4 Department of Biostatistics and Translational Medicine, Medical University of Lodz, 92-215 Lodz, Poland

**Keywords:** cf-DNA, ct-DNA, dd cf-DNA, NIPT, clinical trials, cancer

## Abstract

**Simple Summary:**

Cell-free DNA is present in the plasma and serum of healthy subjects, however, its level is usually much higher in people after physical effort, patients with various diseases such as trauma, sepsis, and also in patients with some cancers. In this concise review, we show the current state of cell-free DNA applications based on clinical trials registered until April 2021. Furthermore, we attempt to summarize a large number of clinical trials and resolve discrepancies raised by these trials. Despite a large number of trials, more studies should include at least 1000 participants and be conducted in large multicenter projects in order to find more accurate results before cfDNA can replace standard diagnostics or often ineffective and hazardous biopsy.

**Abstract:**

We aimed to summarize the current knowledge about the trends in cfDNA application based on the analysis of clinical trials registered until April 2021. International Clinical Trials Registry Platform (ICTRP) and Clinicaltrials.gov were searched with the keywords: “cf-DNA”; “Circulating DNA”; “Deoxyribonucleic Acid”; and “Cell-Free Deoxyribonucleic Acid”. Of 605 clinical trials, we excluded 237 trials, and 368 remaining ones were subject to further analysis. The subject, number of participants, and study design were analyzed. Our scoping review revealed three main trends: oncology (n = 255), non-invasive prenatal diagnostic (n = 48), and organ transplantation (n = 41), and many (n = 22) less common such as sepsis, sport, or autoimmune diseases in 368 clinical trials. Clinical trials are translating theory into clinical care. However, the diagnostic value of cfDNA remains controversial, and diagnostic accuracy still needs to be evaluated. Thus, further studies are necessary until cfDNA turns into a standard in clinical practice.

## 1. Introduction

Circulating DNA not associated with cells is present in the cell-free component of the whole blood, such as plasma and serum, but also in other human body fluids such as urine, saliva, and cerebrospinal fluid [1,2]. The biology of cell-free DNA (cfDNA) is not fully understood, with several possible mechanisms suggested. DNA fragments might be released by apoptosis, necrosis, netosis, or active secretion [3,4]. However, Wang et al. showed that cfDNA concetration did not correlate with amount of apoptotic or necrotic cells, but was mainly released by active secretion [5]. cfDNA is also present in the plasma and serum of healthy subjects, however, its level is usually much higher in people after physical effort, patients with various diseases such as trauma, stroke, burns, sepsis, and also in patients with some cancers. For this reason, cfDNA has become an attractive subject of research as a non-invasive disease biomarker. The analysis includes many structural aspects of total cell-free DNA such as separated fractions of cf nDNA (nuclear) and cf mtDNA (mitochondrial) or cfDNA integrity [2,6,7,8,9]. Formation of cfDNA forms does not occur simultaneously. The level of nDNA corresponds to the general organism’s well-being and increases in the event of pathological damage to cells, whereas cf mtDNA originates from the mitochondrial genome and is released when these organelles are damaged, thus reflecting their condition. cf mtDNA is often elevated in mitochondrial diseases or if stimulated by inflammation, physiological stress, or mechanical injury [10]. Furthermore, cfDNA integrity could be measured, which gives information about the origin of cfDNA, where the physiological death of normal cells is mainly caused by apoptosis and generates DNA fragments of around 180–200 bp in length [11]. Additionally, Tamkovich et al. 2016 found that short fragments (180 bp), compared to long fragments (>8 kbp), were less represented in the plasma of primary breast cancer patients [12].

Rapid progress of cell-free DNA detection methods currently allows determination of a small fraction of tumor-derived cell-free DNA (ctDNA) [1,2]. This type of circulating DNA usually carries mutations or specific modifications, such as methylation originating from cancer tissue. This is a background of minimally invasive liquid biopsy used in early detection disease relapse or monitoring of the effectiveness of radio- and chemotherapy, which could improve cancer management. Apart from oncology, cfDNA is also extensively studied in transplant medicine. Specific donor-derived cell-free DNA (dd-cfDNA) is successfully applied as a non-invasive biomarker for transplant rejection [2,13,14,15], where early detection of allograft rejection is key in preventing allograft loss. cfDNA has already made a huge impact on prenatal medicine, where non-invasive prenatal testing (NIPT) is based on analysis of a fetal component of cell-free DNA in maternal blood. Down syndrome trisomy is detected most frequently. However, NIPT can be used to screen for other common chromosomal aneuploidies, such as trisomy 18 (Edwards syndrome) and trisomy 13 (Patau syndrome) or single-gene mutations [2,16].

The gap between clinical trials and clinical practice is smaller than between scientific studies and clinical practice. Thus, we performed a concise scoping review of databases of clinical trials registered until April 2021 to evaluate current trends in cell-free DNA application.

## 2. Methods

We searched for clinical trials registered in ClinicalTrials.gov and International Clinical Trials Registry Platform (ICTRP) databases for clinical trials registered until April 2021. The subject fields (search terms) were: “*cf-DNA*”; “*Circulating DNA*”; “*Deoxyribonucleic Acid*”; or “*Cell-Free Deoxyribonucleic Acid*”. This search was based upon the assessment structure of the PRISMA statement presented in Supplementary Table S2—PRISMA-ScR checklist. Two independent screenings included both the titles and study descriptions. The trials were reviewed for the type of the studied condition, study design, start date, number of participants, and applied methodology. However, gender, race, or any other population parameters were not considered. Studied conditions were categorized according to the general topic and subtopics, such as type of cancer, and according to the transplanted organ in transplantation medicine.

### Study Selection

**Figure 1:**
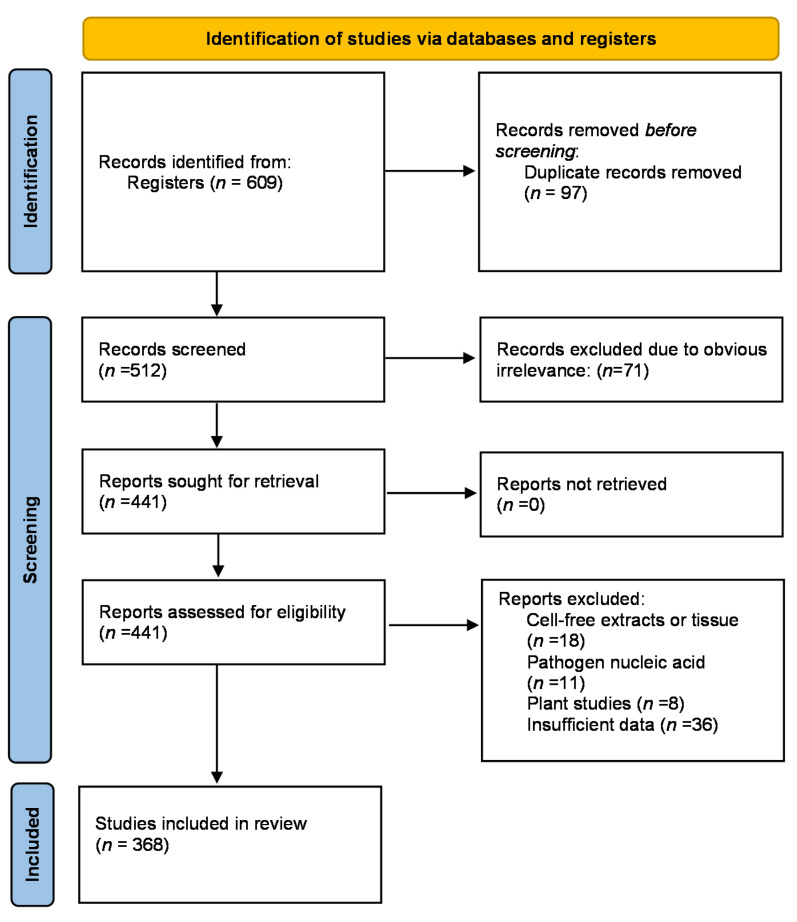
PRISMA flowchart summarizing the identification and selection of studies for inclusion in the present scoping review.

A total of 605 trials were found, and 237 were excluded. The details are presented in Figure 1. The final analysis was done on 368 trials, collected in Supplementary Table S1. The inclusion criteria were: Studies reporting search phrases and their sufficient description. The trials were excluded if they were duplicated or the keyword could not be confirmed. Clinical trials containing phrases such as cell-free extracts, cells, and also pathogen free nucleic acids were excluded to make the review more concise. The flowchart of our review for the purpose of study assessment is shown in Figure 1.

## 3. Results

The results of this analysis include 368 studies in which over 250,000 participants were enrolled. The median sample size was 120 and ranged from less than 50 participants to a relatively large number, i.e., more than 600–1000 participants. Few studies were carried out on more than 15,000 participants, and one study (NCT01511458) included 18,955 participants. Furthermore, an analysis of their age showed that of the total number of over two hundred trials, only 31 included people below the age of 18. All details are shown in Supplementary Table S1.

The study location map was divided symmetrically around the world. About 1/3 took place in the Americas (mostly the US), 1/3 in Europe, and 1/3 in the rest of the world. The number of registered trials analyzed by the start date increased from 17 in the period 2005–2010 to 84 in the period 2011–2015. Finally, it rose to 251 between 2016 and April 2021.

Our analysis of selected clinical trials revealed the presence of all currently studied forms and modifications of cfDNA. The phrase “ctDNA” or “tumor DNA” could be found in 40 projects; 37 clinical trials contained the phrase “liquid biopsy”, 35—the word “fetal”, 21 studies included the phrase “methylation”, five studies contained “cf mtDNA” or “cell-free mitochondrial DNA”, whereas three studies were focused on cfDNA “integrity”. All cell-free DNA forms and modifications used in clinical trials are summarized in Table 1. Most of the available studies have been performed using traditional real-time PCR detection. However, a rising number of selected clinical research is based on novel methods such as NGS (next sequencing generation), ddPCR (digital droplet PCR), CAPPseq (cancer personalized profiling by deep sequencing), PAP (pyrophosphorolysis activated polymerization), BEAMing (beads, emulsion, amplification, and magnetics). Novel methods are characterized by higher feasibility, robustness, and sensitivity.

Notably, cfDNA is studied not only in serum or plasma but also in other bodily fluids such as urine, saliva, or cerebrospinal fluid. Some cancer clinical trials search for a correlation between plasma tumor DNA levels and salivary tumor DNA levels.

Based on the search phrases, the analyzed target genes were: *RAS* in 33, *EGFR* in 27, 14 *BRAF*, *P53* gene in five trials. Moreover, single studies analyzed *NF1*, telomere, or the *IDH1* gene. The details can be found in Supplementary Table S1.

As shown in Table 2, oncology was the most common topic of clinical research (*n* = 256). The number of clinical trials corresponded to the epidemiological frequency of cancer subtypes, so the most common were lung (*n* = 64), colorectal (*n* = 43), breast (*n* = 33), and prostate (*n* = 18) cancers. Moreover, some trials analyzed cancer in general or less common malignancies such as lymphoma or gastric, ovarian, endometrial, and urothelial cancers.

Application of new non-invasive markers, such as dd-cfDNA, for assessment of allograft rejection is becoming a focus of interest. Among 41 phrases containing “dd-cfDNA”; “organ transplant” or “diagnosis of acute rejection”, 24 trials regarded studies on kidney graft rejection, seven regarded lungs, and five—heart transplants.

A total of 50 clinical trials focused on the subject of non-invasive prenatal testing (NIPT). Most of them regarded Down syndrome, but other analyzed disorders also included Edwards syndrome, Patau syndrome, Klinefelter syndrome, Turner syndrome, Di George syndrome. Moreover, rare diseases such as myotonic dystrophy 1, Huntington’s disease, and fragile X syndrome were studied. The details are shown in Supplementary Table S1.

Topics that were difficult to classify were categorized as “other“ and include many different category subjects, such as sepsis, sport, lupus, diabetes, pain, or psychology.

Out of 368 trials, 255 were cancer studies, whereas 112 were non-cancer. An analysis of the recruitment status showed that more than half of the studies were still active. There was a difference between cancer and non-cancer cases. While the number of completed trials was similar, i.e., 30 vs. 39, the recruiting status was more than two-fold higher in cancer studies (109 vs. 47). Furthermore, the total number of all active trials in cancer studies was also higher, i.e., 166 vs. 67 in non-cancer cases. This might indicate a trend towards oncology. All details are shown in Table 3 and Supplementary Table S1.

A total of 368 clinical trials (148 interventional studies and 219 observational studies) for cfDNA were identified. This proportion is similar to that of any other subject of clinical trials. However, there was a difference in proportion between cancer and non-cancer observational studies. Non-cancer cases accounted for less than 30% of the clinical trials, while for observational cases, the number was more than 50%. All details are shown in Table 4.

## 4. Discussion

In this paper, we provide a view on current cfDNA medical application trends. We identified 368 clinical trials registered from April 2005 to April 2021. Out of 368 analyzed studies, 256 regarded cancers. Oncology was the most commonly studied topic. Interestingly, this corresponds to scientific interest and global health problems. Transplant medicine and NIPT were the second most common subject of many clinical trials. However, a brief search of PubMed and Medline databases showed that in comparison to oncology, NIPT and transplant medicine seemed to be discussed less in non-clinical studies on cfDNA.

We conducted a series of subgroup analyses comparing active studies on cancer and non-cancer cases. It appeared that the number of cancer studies was rising (see Table 3). Measurement of cfDNA might improve cancer management [17]. Two decades ago, a technical detection limit allowed only to measure total cfDNA, but nowadays, we are able to detect even a small fraction of tumor-derived cfDNA (ctDNA). A developing tumor might trigger a release of circulating DNA from circulating tumor cells or non-malignant host cells of the immune system [18]. Moreover, cfDNA might serve as a universal marker of pathophysiological changes as a consequence of antineoplastic treatment. Moreover, 40 trials contained the word “ctDNA”, another 37 clinical trials contained the phrase “liquid biopsy”. Currently, molecular diagnostics is mainly based on a small fraction of tumor-derived circulating DNA (ctDNA). Thanks to this, cfDNA-based diagnostic methods should be less invasive compared to traditional biopsy [19]. Furthermore, molecular diagnostics could be faster and more cost-effective. A diagnosis could be made earlier because ctDNA might appear long before tumor metastases give the first symptoms or are detected with traditional diagnostics. Development of detection methods increases their specificity and allows for detection of small fractions of mutated tumor genome. Presence of ctDNA might suggest incomplete resection or cancer metastasis. Thus, ctDNA-based liquid biopsy could monitor the effectiveness of chemo-, immuno-, or radiotherapy [20]. An analysis of ctDNA requires targeting of specific genetic aberrations in circulating DNA, which most commonly are “mutational hot spots”. However, not all tumors present the same molecular signature, besides, even the same histopathological type of tumor can have a diverse mutation pattern. This fact is reflected in a few dozens of clinical trials which target the most common oncogenes or suppressors, such as the EGFR, TP53, BRAF, or the RAS cascade. Moreover, a few dozens of other trials examined specific methylation or applied efficient sequencing methods, such as NGS. This is the next step to personalized cancer management, where individuals will demonstrate precise molecular characteristics of malignant tissues and be administered a targeted therapy for this molecular pattern [21].

Three of the clinical trials found, NCT03284684, NCT03474016, and NCT03892096, analyzed the practical application of DNA integrity in cancer. The pathology of cancer development is associated with increased apoptosis or rather necrosis of the tumor itself or the surrounding tissues, which is reflected in changes in cfDNA integrity [22]. Necrotic DNA fragments are generated more randomly, their size is larger than 200–1000 base pairs, and they can be easily distinguished from shorter fragments with 180–200 base pairs or multiples of this unit in length, produced by physiological apoptosis [11]. The cfDNA integrity index can also be used in monitoring the treatment response. In the case of preoperative chemoradiotherapy, the cfDNA integrity index during treatment was significantly higher in cancer patients [23]. In 2019, Fiala et al. reported that cancer detection sensitivities ranged from 50% to 70%, whereas specificity was between 90% and 95% [24]. In 2018, Cohen et al. applied liquid biopsy (also called CancerSEEK) to 1005 patients with most common and clinically detected nonmetastatic cancers. The sensitivities ranged from 69 to 98% for the detection of five cancer types (of the ovary, liver, stomach, pancreas, and esophagus) for which there are commonly accepted biochemical screening tests. Noteworthy is their specificity, i.e., higher than 99%: only 7 of 812 healthy controls scored positively [25]. According to Sacher and colleagues (2016), an application of ddPCR in plasma of individuals with lung cancer appeared to have a positive predictive value of 100% for EGFR 19 del and 100% for KRAS. The sensitivity of plasma ddPCR was 82% for EGFR 19 del but lower for KRAS, i.e., 64%. Particularly, the sensitivity for EGFR or KRAS was higher in patients with multiple metastatic sites and those with hepatic or bone metastases [26].

The diagnostics of central nervous system (CNS) tumors is usually based on cranial MRI and high-risk brain biopsy. These types of tumors are difficult to detect in the serum or plasma using current liquid biopsy methods. However, tumors release cell-free tumor DNA (cftDNA) into the cerebrospinal fluid (CSF), allowing for potential detection of tumor-associated mutations by CSF sampling. The detection of ctDNA in CSF has been proposed as an alternative to detection in plasma and allows for a prompter and less invasive diagnosis of a CNS tumor. Five ongoing trials focus on this topic (NCT03029065, NCT03711422, NCT04112238, NCT04401774, and NCT03873818). One of them (NCT03711422) focuses on cell-free DNA of CFS in brain metastases due to lung adenocarcinoma [27].

Interestingly, the authors of some clinical trials try to apply cfDNA as a universal cancer marker for no specific type of cancer or for a combination of a few different malignancies. Considering the potential value of early detection in deadly malignancies, further evaluation of this test is justified in prospective population-level studies. A study conducted by Liu et al. (2020) on 6689 participants revealed a panel of over 100,000 informative methylation regions in over 50 cancer types using plasma cfDNA bisulfite sequencing targeting. A classifier was developed and validated for cancer detection and tissue of origin. A targeted methylation analysis of cfDNA allowed to simultaneously detect and localize over 50 cancer types. The cancers were detected across all stages. In stages I–III, the sensitivity was 43.9%; in stages I–IV, the sensitivity was 54.9% (at the specificity of over 99% and a single false positive rate of around 1%). This targeted methylation approach localized the tissue of origin with over 90% accuracy, which will be critical for managing a follow-up care. The authors suggest that cfDNA methylation patterns detected more than 50 cancer types across the stages [28].

Furthermore, the ratio between the number of still active and recruiting trials and inactive or finished ones suggests that oncology is not only the main but also a steadily growing trend.

Assessment of allograft rejection, made in 61 trials, is the second most common trend in cfDNA clinical trials. The presence of donor-derived cell-free DNA (dd-cfDNA) in the blood or urine of transplant recipients correlates with the risk of rejection. At first, dd-cfDNA was based on Y-chromosome detection. However, it was limited by male-to-female transfers. Currently, detection of dd-cfDNA requires prior knowledge of donor and recipient genotypes, then, the accuracy of current methods allows to distinguish even a single nucleotide polymorphism. Similarly, in oncology, minimally invasive diagnostics might potentially replace traditional tissue biopsies. Furthermore, similarly to cancer, it requires highly sensitive detection methods which will be able to detect less than 1% of DNA from the total cfDNA fraction [29].

Schutz et al. (2019) analyzed liver transplants and observed that the diagnostic sensitivity and specificity were 90.3% and 92.9%, respectively, for graft-derived circulating DNA [30]. Sayah et al. analyzed lung transplant patients and suggested that dd-cfDNA-sensitivity was 73.1%, whereas its specificity was 52.9% [31]. Furthermore, in heart transplants, a diagnosis can be predicted with 92% sensitivity and 75% specificity [32]. By assessing donor-derived cell-free DNA in a kidney transplant, Huang et al. observed that a kidney injury can be detected with 100% sensitivity and 71.8% specificity. However, the dd-cfDNA test did not discriminate cellular rejection from no rejection among organ recipients [33]. Sigdel et al. carried out a study using NGS in 300 plasma samples collected from patients after a kidney transplant. In this retrospective analysis, the method enabled to obtain more than 80% sensitivity and more than 70% specificity in detecting dd-cfDNA levels [34]. It is assumed that the probability of acute rejection increases when the dd-cfDNA level exceeds 1%. Gielis et al. (2020) showed with 107 kidney transplant recipients that dd-cfDNA rises above a threshold value of 0.88% were significantly associated with the occurrence of episodes of acute rejection [35]. In a study with 87 patients, Knutgen et al. (2021) showed that with a dd-cfDNA cut-off of 0.35%, sensitivity and specificity of dd-cfDNA for cardiac rejection were 0.76 and 0.83, respectively [36]. In lung transplantation, the optimal threshold for dd-cfDNA for aggregated rejection events representing allograft injury was determined as 0.85%, with sensitivity of 55.6% and specificity of 75.8% [37]. In tBPAR (biopsy-proven acute rejection requiring treatment after liver transplantation), the receiver operator characteristic curve analysis (ROC) showed that dd-cfDNA values reached 98.8% and were higher than in routine liver function tests [38].

The three of four most commonly transplanted organs, i.e., the kidney, liver, heart, and lung, are extensively studied in clinical trials. In our analysis, the only exception seemed to be liver transplantation, presented only in one study. Although many studies have confirmed the correlation of dd-cfDNA with both the biopsy and the rejection diagram, this procedure has still not been approved by the American Food and Drug Administration or the European Medicines Agency. Probably, both the threshold value and kinetics need to be clarified.

Prenatal cell-free screening, also known as non-invasive prenatal screening, is a screening method applied for the purpose of detection of certain genetic abnormalities in a fetus. Although NIPT is the third method in relation to the total number of registered trials, it seems to be about to be officially registered. In contrast to standard diagnostics, it does not pose the risk of miscarriage. DNA from the mother and the fetus is extracted from maternal blood and screened for a potential and increased chance of specific chromosomal problems, such as Down syndrome, trisomy 13, and trisomy 18, and also single-gene disorders. Moreover, it can also be used to screen for fetal sex [39]. However, about 1% to 5% of prenatal cell-free DNA screening tests do not yield any result, possibly due to an insufficient amount of DNA. The screening is not effective for women pregnant with multiples, for mothers with a disturbed body mass index, or via a donor egg [40]. Some trials are focused on just one aneuploidy, such as 21 trisomy (Down syndrome) whereas others combine screening of aneuploidy or examination of single-gene aberrations. Taylor-Philips et al. (2016), in a systematic review and meta-analysis based on a cohort of 112,669, emphasize high specificity and sensitivity of such prenatal diagnostics [41]. The pooled sensitivity for Down syndrome is over 99.3%, whereas for Edwards syndrome, it is 97.4% [41].

There is also a possibility of occurrence of false positive and false negative results due to a lab error. Since the source of fetal cell-free DNA is the placenta, mistakes might thus be caused by confined placental mosaicism. Additionally, an early fetal demise may also result in a false positive result. False negative results may be obtained when the fetus appears to have trisomy. However, the placental cell line may undergo a trisomy rescue event where the predominant cell line of the placenta is chromosomally normal. Moreover, any confounding factor, such as chromosomal abnormality in the maternal cell line (mosaic or otherwise), may result in an inaccurate result [42].

Both pregnant women and obstetric care providers are aware of rapid advances in NIPT and appear to look forward to its clinical introduction. Therefore, there is some urgency to perform large-scale properly conducted clinical evaluation studies. However, commercial genetic testing offering paid tests may disturb these studies by pushing those who buy such tests out of a disease detection procedure. In this context, we should also thoroughly evaluate all ethical and social implications of genetic tests. However, progress in diagnostic accuracy and cost-effectiveness based on large-scale clinical trials is still needed. What is important is the fact that the UK National Screening Committee officially accepted this form of diagnostics in most common aneuploidies in first-trimester pregnancies.

A large number of clinical trials bearing the category “Other” suggest a huge diversity of subjects. Only four trials (ChiCTR-OOC-15006382, NCT00919685, NCT03356249, and NCT04189549) applied cfDNA as a marker of sepsis. The details are shown in Table 5.

Sepsis, a potentially life-threatening medical condition associated with an infection, is an important problem in emergency patients or patients affected by infectious diseases, such as COVID-19. The inflammatory stress significantly increases many markers as well as the cell-free DNA level. Patients with sepsis demonstrate twenty-two times higher levels of cfDNA compared to those of non-septic patients. In addition, it was found that the level of cell-free DNA allows to divide sepsis patients admitted to the emergency room into survivors and non-survivors [43,44].

Only a few trials explore the application of cfDNA in sport, despite the fact that the exercise-induced increase is very high. It seems that cf n-DNA could be more sensitive in reflecting acute exercise-induced changes in the human body than other markers, such as AST and ALT CK or CRP [45]. Therefore, measurements of exercise-related variations of cfDNA can be used for monitoring of training load or diagnosis and prevention of overtraining syndrome [46]. The first of the “sport-trials” examines the aspect of sport and psychology (NCT02874833), whereas another one deals with overtraining (NCT03833973). In the context of billions of dollars currently being spent on professional sport, improved diagnostics seems to be very promising.

Apart from nDNA, cfDNA subtractions are also extensively studied. mtDNA is often associated with mitochondrial disorders, such as neurodegenerative or autoimmune disorders. In order to obtain a higher diagnostic value, many studies combine different aspects of cfDNA structure [47,48,49]. Two trials deal with infertility in the context of embryo quality. A single clinical trial on cfDNA is performed in diabetes (NCT02898467), cardiac sarcoidosis (NCT03858777), and preeclampsia (pregnancy complication; NCT03067298). Moreover, one has been carried out to detect the paraneoplastic syndrome of the nervous system (NCT04454853) and spinal muscular atrophy (NCT04690998). Another study deals with molecular testing of neurodegeneration (NCT03938909), where cytokines and cf mtDNA could be applied as an efficient marker of a disease. cf mtDNA is also studied in acute inflammatory response to psychological stress, while cell-free DNA in psychology is suggested as a putative marker of depression or psychological stress [50]. Two studies combine sport and cancer diagnostics (Supplementary Table S1). Interestingly, in some cancers, such as PTC or breast cancer, changes in mtDNA are not associated with cfnDNA. When nDNA is increasing, cf mtDNA is decreasing [7,48].

A few studies could not be clearly classified because they mixed different conditions, such as lupus and exercise, cancer and exercise or pulmonary embolism, myocardial infarction, and autoimmune disease.

Traditional detection of cfDNA using real-time PCR seems to be “incongruous with the times”. However, novel methods such as NGS, ddPCR, CAPPseq, PAP, BEAMing, and Microarray CGH analysis are becoming commonplace. These novel methods are characterized by higher feasibility, robustness, and sensitivity. Nevertheless, we are unable to present the full picture, since not all descriptions presented a detailed methodology.

cfDNA is not measured independently. Usually, it is examined in the context of other traditional biochemical markers, such as cytokines, CRP, CK, or hormones. cfDNA should be used along with standard diagnostic methods, such MRI, CT, or traditional biopsy. On the other hand, cfDNA detection might be combined with other molecular markers such as mRNA, miRNA, or immunophenotyping to improve diagnostic value.

Due to a huge number of different forms, modifications, and putative application, a gradual evolution, rather than revolutionary changes, can be expected. qPCR is a commonly accepted detection method, even though clinical research was conducted using new methodological trends characterized with unprecedented throughput, such as ddPCR, which allows to quantify rare targets in a complex background and exhibits high tolerance to PCR inhibitors as well as enables to detect a small fold change.

Furthermore, the selection of a control group, including randomization, is a critical decision in clinical trial design. The control group provides information on potential consequences for participants if they were not treated or alternatively treated. A brief assessment suggests that the study design is logical and made in compliance with general rules of clinical research. Sample size is an important element of a trial because cohorts that are too large are uneconomical. However, what is even more important is the fact groups that are too small could result in obtaining uncertain outcomes [51]. Many factors influence sample size. In our analysis of more than 70 trials, over 500 volunteers were going to be recruited, which seems to be a reliable number.

In an observational study, investigators assess health outcomes in participants according to a research plan or protocol. In contrast to interventional studies, they do not receive any therapeutic intervention. In observational studies, investigators often compare the difference of outcomes for two groups, which is a better option in non-cancer studies. A rising number of clinical trials, from 17 in 2005–2010 to over 250 in 2015–2021, shows a larger interest in cell-free DNA application in clinical practice. To sum up, a comparison of cancer research with non-cancer research indicates that there is a shift of active trials towards oncology research.

An analysis of clinical trials by recruitment status showed that over 220 trials are still active or recruiting, while almost 170 have been completed or suspended. This indicates that cell-free DNA, despite several dozen years of scientific research, is now being transferred to the clinical research phase. More studies should be conducted in large multicenter projects and include more than 1000 participants. This will enable to obtain more accurate results before cfDNA can replace biopsy, which is often ineffective and hazardous.

Study limitations:

(1) Projects with high application potential are subject to clinical trials, which does not necessarily reflect the real scientific/industrial interest.
(2) Only two biggest international clinical trial databases were used. Minor national databases were not taken into account.

## 5. Conclusions

Herein we have noticed:

(1) The analysis was based on 368 trials attended by over 250,000 participants, where 233 are still active or recruiting.
(2) The vast majority of 255 clinical trials are on oncology, 48 on NIPT, and 41 on transplant medicine.
(3) The projects are performed using modern methods.

## Figures and Tables

**Table 1:** Cell-free DNA forms and modifications used in clinical trials.

Cell-Free DNA	Abbreviation	Application
Total cfDNA	cfDNA, ccfDNA	General marker of physiological well-being, elevated in sepsis or under stress, temporarily increases during exercise
Circulating cell-free DNA		
Serum/Plasma cell-free DNA		
Circulating free DNA		
Tumoral plasma DNA	ctDNA	Used in cancer therapy management, marker of cancer relapse or metastasis
(Circulating tumoral DNA)		
Donor Derived cell-free DNA	dd-cfDNA	Used in detection of allograft rejection in transplantation
(Donor specific cell-free DNA)	dscfDNA	
cell-free fetal DNA	cff-DNA	Used in non-invasive prenatal diagnostics, usually aneuploidy
circulating cell-free fetal DNA	ccff-DNA	
Mitochondrial cfDNA	cf mtDNA	Releases during mitochondrial dysfunction, elevated under physiological stress or in some cancers
Circulating mitochondrial DNA		
Methylated cell-free DNA	m-cfDNA	Mostly applied in oncology as a marker for cancer screening. Could be done specifically for a selected gene or globally
cfDNA integrity index	cfDI	Allows to distinguish if cfDNA is released from apoptotic or necrotic cells

**Table 2:** Conditions targeted by clinical trials.

Condition	Specific Condition
Cancer (*n* = 255)	Lung Cancer (*n* = 64)
	Colorectal cancer (*n* = 43)
	Breast (*n* = 33)
	Prostate (*n* = 18)
	Other (including all cancer diagnoses) (*n* = 97)
NIPT (*n* = 50)	Prenatal Testing (*n* = 50)
Organ Transplant (*n* = 41)	Kidney (*n* = 24)
	Lung (*n* = 7)
	Heart (*n* = 5)
	Other (*n* = 5)
Other (*n* = 22)	Sepsis (*n* = 4)
	Other (*n* = 18)

**Table 3:** Clinical trials’ recruitment status, including cancer vs. non-cancer comparison.

Recruitment Status	Number of Studies in Total	Number of Studies Cancer	Number of Studies Non-Cancer
Recruiting	156	109	47
Completed	69	39	30
Not yet recruiting	55	41	14
Suspended/Unknown status	52	40	12
Active, not recruiting	22	16	6
Terminated	8	7	1
Enrolling by invitation	3	1	2
Withdrawn	3	3	0

**Table 4:** Clinical trial study design, including cancer vs. non-cancer studies comparison.

Type	Number of Studies in Total	Number of Studies Cancer	Number of Studies Non-Cancer
Interventional	148	114	34
Observational Study	219	141	78

**Table 5:** Clinical trial study in sepsis: ID number, title, status, primary outcomes.

ID Number	Title	Status	Primary Outcomes
ChiCTR-OOC-15006382	Prognostic value of circulating cell-free DNA in patientsadmitted to Emergency Intensive Care Unit (EICU)	Active/recruiting	Serum cfDNA level; Prognose;
NCT03356249	Next-Generation Sequencing Diagnostics of Bacteremia in Sepsis (Next GeneSiS-Trial)	Active/Recruiting	Sensitivity; Specificity; Positive predictive value
NCT00919685	Investigations of New Markers in Patients with Shock	Completed	Demonstrate the superiority of at least one of 3 markers (HIF, MPs, cDNA) with regard to plasma lactate level for evaluating the treatment response in patients with shock
NCT04189549	Preclinical Detection of Sepsis Early in Hospitalized Patients Following Surgery, Injury or Severe Illness (Pre-SEPSIS Trial)	Not yet recruiting	Clinical development of sepsis

## Data Availability

The data presented in this study are openly available in public databases.

## Supplementary Materials

The following are available online at https://www.mdpi.com/article/10.3390/biology10090906/s1, Table S1: Clinical Trial analysis, Table S2: PRISMA-ScR checklist.

## References

[1] Ponti G., Manfredini M., Tomasi A. (2019). Non-blood sources of cell-free DNA for cancer molecular profiling in clinical pathology and oncology. Crit. Rev. Oncol..

[2] Bronkhorst A.J., Ungerer V., Diehl F., Anker P., Dor Y., Fleischhacker M., Gahan P.B., Hui L., Holdenrieder S., Thierry A.R. (2021). Towards systematic nomenclature for cell-free DNA. Qual. Life Res..

[3] Kustanovich A., Schwartz R., Peretz T., Grinshpun A. (2019). Life and death of circulating cell-free DNA. Cancer Biol. Ther..

[4] Grabuschnig S., Bronkhorst A.J., Holdenrieder S., Rodriguez I.R., Schliep K.P., Schwendenwein D., Ungerer V., Sensen C.W. (2020). Putative Origins of Cell-Free DNA in Humans: A Review of Active and Passive Nucleic Acid Release Mechanisms. Int. J. Mol. Sci..

[5] Wang W., Kong P., Ma G., Li L., Zhu J., Xia T., Xie H., Zhou W., Wang S. (2017). Characterization of the release and biological significance of cell-free DNA from breast cancer cell lines. Oncotarget.

[6] Zachariah R.R., Schmid S., Buerki N., Radpour R., Holzgreve W., Zhong X. (2008). Levels of Circulating Cell-Free Nuclear and Mitochondrial DNA in Benign and Malignant Ovarian Tumors. Obstet. Gynecol..

[7] Kohler C., Radpour R., Barekati Z., Asadollahi R., Bitzer J., Wight E., Bürki N., Diesch C., Holzgreve W., Zhong X.Y. (2009). Levels of plasma circulating cell free nuclear and mitochondrial DNA as potential biomarkers for breast tumors. Mol. Cancer.

[8] Salvianti F., Giuliani C., Petrone L., Mancini I., Vezzosi V., Pupilli C., Pinzani P. (2017). Integrity and Quantity of Total Cell-Free DNA in the Diagnosis of Thyroid Cancer: Correlation with Cytological Classification. Int. J. Mol. Sci..

[9] Li L., Hann H.-W., Wan S., Hann R.S., Wang C., Lai Y., Ye X., Evans A., Myers R.E., Ye Z. (2016). Cell-free circulating mitochondrial DNA content and risk of hepatocellular carcinoma in patients with chronic HBV infection. Sci. Rep..

[10] Sharma P., Sampath H. (2019). Mitochondrial DNA Integrity: Role in Health and Disease. Cells.

[11] Stawski R., Walczak K., Perdas E., Wlodarczyk A., Sarniak A., Kosielski P., Meissner P., Budlewski T., Padula G., Nowak D. (2019). Decreased integrity of exercise-induced plasma cell free nuclear DNA—Negative association with the increased oxidants production by circulating phagocytes. Sci. Rep..

[12] Tamkovich S.N., Kirushina N.A., Voytsitskiy V.E., Tkachuk V.A., Laktionov P.P. (2016). Features of Circulating DNA Fragmentation in Blood of Healthy Females and Breast Cancer Patients. Adv. Exp. Med. Biol..

[13] Knight S.R., Thorne A., Faro M.L.L. (2019). Donor-specific Cell-free DNA as a Biomarker in Solid Organ Transplantation. A Systematic Review. Transplantation.

[14] Garg N., Hidalgo L.G., Aziz F., Parajuli S., Mohamed M., Mandelbrot D.A., Djamali A. (2020). Use of Donor-Derived Cell-Free DNA for Assessment of Allograft Injury in Kidney Transplant Recipients During the Time of the Coronavirus Disease 2019 Pandemic. Transplant. Proc..

[15] Dengu F. (2020). Next-generation sequencing methods to detect donor-derived cell-free DNA after transplantation. Transplant. Rev..

[16] Goldwaser T., Klugman S. (2018). Cell-free DNA for the detection of fetal aneuploidy. Fertil. Steril..

[17] Stewart C.M., Tsui D.W. (2018). Circulating cell-free DNA for non-invasive cancer management. Cancer Genet..

[18] Gahan P.B., Anker P., Stroun M. (2008). Metabolic DNA as the Origin of Spontaneously Released DNA?. Ann. N. Y. Acad. Sci..

[19] Perdas E., Stawski R., Nowak D., Zubrzycka M. (2018). Potential of Liquid Biopsy in Papillary Thyroid Carcinoma in Context of miRNA, BRAF and p53 Mutation. Curr. Drug Targets.

[20] Osumi H., Shinozaki E., Yamaguchi K. (2020). Circulating Tumor DNA as a Novel Biomarker Optimizing Chemotherapy for Colorectal Cancer. Cancers.

[21] Oliveira K.C.S., Ramos I.B., Silva J.M.C., Barra W.F., Riggins G.J., Palande V., Pinho C.T., Frenkel-Morgenstern M., Santos S.E., Assumpcao P.P. (2020). Current Perspectives on Circulating Tumor DNA, Precision Medicine, and Personalized Clinical Management of Cancer. Mol. Cancer Res..

[22] Fleischhacker M., Schmidt B. (2007). Circulating nucleic acids (CNAs) and cancer—A survey. Biochim. Biophys. Acta (BBA) Rev. Cancer.

[23] Kumar M., Choudhury Y., Ghosh S.K., Mondal R. (2018). Application and optimization of minimally invasive cell-free DNA techniques in oncogenomics. Tumor Biol..

[24] Fiala C., Diamandis E.P. (2019). New approaches for detecting cancer with circulating cell-free DNA. BMC Med..

[25] Cohen J.D., Li L., Wang Y., Thoburn C., Afsari B., Danilova L., Douville C., Javed A.A., Wong F., Mattox A. (2018). Detection and localization of surgically resectable cancers with a multi-analyte blood test. Science.

[26] Sacher A., Paweletz C., Dahlberg S., Alden R.S., O’Connell A., Feeney N., Mach S.L., Jänne P.A., Oxnard G.R. (2016). Prospective Validation of Rapid Plasma Genotyping for the Detection ofEGFRandKRASMutations in Advanced Lung Cancer. JAMA Oncol..

[27] Rimelen V., Ahle G., Pencreach E., Zinniger N., Debliquis A., Zalmaï L., Harzallah I., Hurstel R., Alamome I., Lamy F. (2019). Tumor cell-free DNA detection in CSF for primary CNS lymphoma diagnosis. Acta Neuropathol. Commun..

[28] Liu M., Oxnard G., Klein E., Swanton C., Seiden M., Smith D., Richards D., Yeatman T.J., Cohn A.L., Lapham R. (2020). Sensitive and specific multi-cancer detection and localization using methylation signatures in cell-free DNA. Ann. Oncol..

[29] Martuszewski A., Paluszkiewicz P., Król M., Banasik M., Kepinska M. (2021). Donor-Derived Cell-Free DNA in Kidney Transplantation as a Potential Rejection Biomarker: A Systematic Literature Review. J. Clin. Med..

[30] Schütz E., Fischer A., Beck J., Harden M., Koch M., Wuensch T., Stockmann M., Nashan B., Kollmar O., Matthaei J. (2017). Graft-derived cell-free DNA, a noninvasive early rejection and graft damage marker in liver transplantation: A prospective, observational, multicenter cohort study. PLoS Med..

[31] Sayah D., Weigt S.S., Ramsey A., Ardehali A., Golden J., Ross D.J. (2020). Plasma Donor-derived Cell-free DNA Levels Are Increased During Acute Cellular Rejection After Lung Transplant: Pilot Data. Transplant. Direct.

[32] Richmond M.E., Zangwill S.D., Kindel S.J., Deshpande S.R., Schroder J.N., Bichell D.P., Knecht K.R., Mahle W.T., Wigger M.A., Gaglianello N.A. (2020). Donor fraction cell-free DNA and rejection in adult and pediatric heart transplantation. J. Heart Lung Transplant..

[33] Huang E., Sethi S., Peng A., Najjar R., Mirocha J., Haas M., Vo A., Jordan S.C. (2019). Early clinical experience using donor-derived cell-free DNA to detect rejection in kidney transplant recipients. Am. J. Transplant..

[34] Sigdel T.K., Archila F.A., Constantin T., Prins S.A., Liberto J., Damm I., Towfighi P., Navarro S., Kirkizlar E., Demko Z. (2018). Optimizing Detection of Kidney Transplant Injury by Assessment of Donor-Derived Cell-Free DNA via Massively Multiplex PCR. J. Clin. Med..

[35] Gielis E.M., Ledeganck K.J., Dendooven A., Meysman P., Beirnaert C., Laukens K., De Schrijver J., Van Laecke S., Van Biesen W., Emonds M.-P. (2019). The use of plasma donor-derived, cell-free DNA to monitor acute rejection after kidney transplantation. Nephrol. Dial. Transplant..

[36] Knüttgen F., Beck J., Dittrich M., Oellerich M., Zittermann A., Schulz U., Fuchs U., Knabbe C., Schütz E., Gummert J. (2021). Graft-Derived Cell-Free DNA as a Noninvasive Biomarker of Cardiac Allograft Rejection: A Cohort Study on Clinical Validity and Confounding Factors. Transplantation.

[37] Khush K.K., De Vlaminck I., Luikart H., Ross D.J., Nicolls M.R. (2021). Donor-derived, cell-free DNA levels by next-generation targeted sequencing are elevated in allograft rejection after lung transplantation. ERJ Open Res..

[38] Goh S.K., Do H., Testro A., Pavlovic J., Vago A., Lokan J., Jones R.M., Christophi C., Dobrovic A., Muralidharan V. (2019). The Measurement of Donor-Specific Cell-Free DNA Identifies Recipients With Biopsy-Proven Acute Rejection Requiring Treatment After Liver Transplantation. Transplant. Direct.

[39] Carbone L., Cariati F., Sarno L., Conforti A., Bagnulo F., Strina I., Pastore L., Maruotti G.M., Alviggi C. (2021). Non-Invasive Prenatal Testing: Current Perspectives and Future Challenges. Genes.

[40] Kruckow S., Schelde P., Hatt L., Ravn K., Petersen O.B., Uldbjerg N., Vogel I., Singh R. (2019). Does Maternal Body Mass Index Affect the Quantity of Circulating Fetal Cells Available to Use for Cell-Based Noninvasive Prenatal Test in High-Risk Pregnancies?. Fetal Diagn. Ther..

[41] Taylor-Phillips S., Freeman K., Geppert J., Agbebiyi A., Uthman A.O., Madan J., Clarke A., Quenby S., Clarke A. (2016). Accuracy of non-invasive prenatal testing using cell-free DNA for detection of Down, Edwards and Patau syndromes: A systematic review and meta-analysis. BMJ Open.

[42] McCullough R.M., Almasri E.A., Guan X., Geis J.A., Hicks S.C., Mazloom A.R., Deciu C., Oeth P., Bombard A.T., Paxton B. (2014). Non-Invasive Prenatal Chromosomal Aneuploidy Testing—Clinical Experience: 100,000 Clinical Samples. PLoS ONE.

[43] Clementi A., Virzì G.M., Brocca A., Pastori S., De Cal M., Marcante S., Granata A., Ronco C. (2016). The Role of Cell-Free Plasma DNA in Critically Ill Patients with Sepsis. Blood Purif..

[44] Long Y., Zhang Y., Gong Y., Sun R., Su L., Lin X., Shen A., Zhou J., Caiji Z., Wang X. (2016). Diagnosis of Sepsis with Cell-free DNA by Next-Generation Sequencing Technology in ICU Patients. Arch. Med. Res..

[45] Stawski R., Walczak K., Kosielski P., Meissner P., Budlewski T., Padula G., Nowak D. (2017). Repeated bouts of exhaustive exercise increase circulating cell free nuclear and mitochondrial DNA without development of tolerance in healthy men. PLoS ONE.

[46] Breitbach S., Tug S., Simon P. (2012). Circulating Cell-Free DNA. Sports Med..

[47] Dauber E., Kollmann D., Kozakowski N., Rasoul-Rockenschaub S., Soliman T., Berlakovich G.A., Mayr W.R. (2019). Quantitative PCR of INDELs to measure donor-derived cell-free DNA—A potential method to detect acute rejection in kidney transplantation: A pilot study. Transpl. Int..

[48] Perdas E., Stawski R., Kaczka K., Nowak D., Zubrzycka M. (2019). Altered levels of circulating nuclear and mitochondrial DNA in patients with Papillary Thyroid Cancer. Sci. Rep..

[49] Chang C.-C., Chiu P.-F., Wu C.-L., Kuo C.-L., Huang C.-S., Liu C.-S. (2019). Urinary cell-free mitochondrial and nuclear deoxyribonucleic acid correlates with the prognosis of chronic kidney diseases. BMC Nephrol..

[50] Trumpff C., Marsland A.L., Basualto-Alarcón C., Martin J.L., Carroll J.E., Sturm G., Vincent A.E., Mosharov E.V., Gu Z., Kaufman B.A. (2019). Acute psychological stress increases serum circulating cell-free mitochondrial DNA. Psychoneuroendocrinology.

[51] Day S., Jonker A.H., Lau L.P.L., Hilgers R.-D., Irony I., Larsson K., Roes K.C., Stallard N. (2018). Recommendations for the design of small population clinical trials. Orphanet J. Rare Dis..

